# Epidemiological characteristics of co-infection between porcine epidemic diarrhea virus (PEDV) and other pathogens: a meta-analysis and systematic review

**DOI:** 10.1186/s40813-026-00509-1

**Published:** 2026-04-17

**Authors:** Hong Zou, Zheng Niu, Yi Fan, Guihua Fu, Gan Luo, Zhiping Mu

**Affiliations:** 1 College of Animal Science & Technology, Chongqing Three Gorges Vocational College, Chongqing, 404100 China; 2 College of Veterinary Medicine, Northwest A & F University, Xianyang, 712000 China; 3 Wanzhou Center for Animal Husbandry Industry Development of Chongqing, Chongqing, 404100 China

**Keywords:** Porcine epidemic diarrhea virus, Co-infection, Epidemiology, Spatiotemporal distribution

## Abstract

**Background:**

Currently, despite numerous local epidemiological investigations, knowledge of porcine epidemic diarrhea virus (PEDV) coinfections remains fragmented. Most existing studies are restricted to individual regions or specific farms with limited sample sizes, and a comprehensive synthesis of PEDV coinfection epidemiology at the global level remains lacking. Consequently, the overall prevalence of PEDV coinfections, predominant pathogen combinations, and their spatiotemporal dynamics remain poorly defined. Here, we systematically characterize PEDV coinfections with other pathogens and investigate their spatiotemporal distribution across China.

**Methods:**

This study strictly followed the PRISMA guidelines. Four major databases (CNKI, PubMed, Web of Science, and Scopus) were systematically searched for cross-sectional epidemiological studies on PEDV coinfections published from database inception to February 1, 2026. In total, 60 eligible studies comprising 49,455 samples were included. Meta-analysis, subgroup analysis, meta-regression, and spatiotemporal stratification were conducted.

**Results:**

The pooled coinfection rate of PEDV with other pathogens was 12% (95% CI: 0.09–0.16). Funnel plots, together with Egger’s and Begg’s tests, indicated no significant publication bias, and sensitivity analyses confirmed the robustness of the findings. A total of 121 coinfection patterns were identified, with double infections predominating (83.47%), followed by triple (14.88%) and quadruple infections (1.65%). Among double infections, PEDV-PDCoV was the most common combination (23.1%), followed by PEDV-TGEV (13.2%) and PEDV-PoRV (10.7%). Subgroup analyses demonstrated that farm size and coinfection type were the primary sources of heterogeneity (both *P* < 0.001). Spatiotemporal analyses across five geographical regions of China revealed pronounced heterogeneity in PEDV coinfections. The eastern (ES = 12.40%, I² = 74.9%) and northwestern (ES = 12.90%, I² = 75.4%) regions exhibited the highest coinfection rates and the most complex pathogen profiles. In contrast, the central-southern region showed the lowest coinfection rate (ES = 2.50%, I² = 50.6%), suggesting effective PEDV control. The northern region displayed stable epidemic characteristics, with coinfections exclusively involving PDCoV (ES = 6.60%, I² = 0%), whereas the southwestern region showed a declining trend in single infections accompanied by an increase in coinfections (ES = 5.50%, I² = 68.2%).

**Conclusion:**

This study characterizes the epidemiological features, predominant pathogen combinations, and China’s regional spatiotemporal patterns of porcine epidemic diarrhea virus (PEDV) co-infections. However, these findings should be interpreted with caution, as potential detection bias exists; the reported co-infection rates and pathogen profiles were influenced by heterogeneity in the panels of target pathogens tested across the included studies. Despite this limitation, our results provide valuable data to support the development of region-specific, precision-based diagnostic and prevention strategies for PEDV-associated diseases.

**Systematic review registration:**

Open Science Framework (10.17605/OSF.IO/9UY8F).

## Introduction

Porcine epidemic diarrhea (PED) is a highly contagious enteric disease caused by porcine epidemic diarrhea virus (PEDV) and is clinically characterized by vomiting, acute watery diarrhea, dehydration, and high mortality [1]. Newborn piglets are particularly susceptible, with mortality rates reaching 80–100% [2]. In recent years, the epidemiological characteristics of PEDV have changed markedly [3]. The proportion of PEDV single infections has declined, whereas coinfections with other pathogens have become increasingly common in clinical settings [4]. Coinfection, defined as the simultaneous or sequential infection of a host by two or more pathogens, is influenced by multiple factors, including pathogen interactions, host immune status, husbandry conditions, and disease control measures [5]. Previous studies have shown that coinfections of PEDV with pathogens such as porcine deltacoronavirus (PDCoV) [6], transmissible gastroenteritis virus (TGEV) [7], porcine rotavirus (PoRV) [8], and porcine circovirus (PCV) [9] exacerbate clinical symptoms, prolong disease duration, increase mortality, and complicate diagnosis and control [10]. These effects are mediated through mechanisms including disruption of intestinal mucosal barrier integrity, interference with host immune responses, and enhanced pathogenicity [11]. For instance, PEDV-PDCoV coinfection can synergistically damage intestinal epithelial cells, resulting in more severe diarrhea and dehydration [12]. Moreover, complex multi-pathogen interactions may impair vaccine-induced protective immunity [13].

Despite the abundance of local epidemiological investigations on PEDV coinfections worldwide, existing studies have notable limitations [4, 14–17]. Most investigations have been confined to single regions or individual farms, with limited sample sizes, and lack a systematic, integrative analysis of the global epidemiological characteristics of PEDV coinfections, particularly across different regions of China. As a result, the overall incidence, predominant coinfection patterns, and underlying epidemic features remain poorly understood. Current research provides fragmented evidence regarding factors influencing PEDV coinfections and has failed to perform systematic subgroup analyses based on key variables, such as farm scale, production systems, sampling periods, and geographical regions. This has hindered the identification of core determinants of coinfection occurrence. Moreover, studies addressing the spatiotemporal evolutionary dynamics of PEDV coinfections are scarce, limiting our understanding of temporal trends and regional shifts in coinfection patterns. The diversity of coinfection types and regional heterogeneity in dominant combinations have also not been comprehensively summarized.

Against this backdrop, this study strictly adhered to the Preferred Reporting Items for Systematic Reviews and Meta-Analyses (PRISMA) statement. A systematic search was conducted across four major databases (CNKI, PubMed, Web of Science, and Scopus), and cross-sectional epidemiological studies on PEDV coinfections published from database inception to February one, 2026, were included. This study investigated the overall prevalence, primary coinfection types, and dominant combinations of PEDV coinfections with other pathogens. Subgroup analyses and meta-regression were conducted to assess the effects of key factors, including farm size, coinfection type, geographical region, and sampling time, on coinfection rates and sources of heterogeneity. Spatiotemporal subgroup analyses across five geographical regions of China were performed to elucidate the regional distribution characteristics and temporal evolutionary patterns of PEDV coinfections. These findings provide valuable insights for the diagnosis, prevention, and control of PEDV coinfections.

## Methods

### Study search and inclusion criteria

This study was conducted in accordance with the PRISMA guidelines to systematically retrieve and screen relevant literature, to clarify the epidemiological characteristics of co-infection between PEDV and other pathogens. Literature retrieval covered the period from database inception to February one, 2026. To ensure comprehensive coverage of relevant evidence, both the Chinese database CNKI and the English databases PubMed, Web of Science, and Scopus were searched. A high-sensitivity search strategy was developed based on the research topic as follows: CNKI: (Porcine epidemic diarrhea virus OR PEDV) AND (Co-infection OR Mixed infection); PubMed: PEDV[All Fields] AND Coinfection[All Fields] AND Swine[All Fields]; Web of Science: TS=(PEDV) AND TS=(Coinfection OR “Mixed infection”) AND TS=(Swine OR Piglets); Scopus: PEDV AND (Coinfection OR “Mixed infection”) AND Swine. Literature screening was independently performed by two researchers according to predefined inclusion and exclusion criteria and consisted of two stages: initial screening of titles and abstracts, followed by full-text review. Discrepancies were resolved through discussion, with arbitration by a third researcher when necessary.

Inclusion criteria were as follows: (1) cross-sectional epidemiological survey design; (2) clear reporting of study area and geographical information; (3) swine herds as study subjects, with explicit detection and reporting of PEDV co-infection with other pathogens and available quantitative data (e.g., total sample size and number of co-infected samples); (4) detection of PEDV and co-infecting pathogens using molecular methods such as RT-PCR or qRT-PCR with reproducible methodological descriptions; and (5) availability of full text.

Exclusion criteria included: (1) non-epidemiological studies such as reviews, case reports, in vitro experiments, and animal challenge studies; (2) studies reporting only PEDV mono-infection without co-infection data; and (3) studies with unavailable or ambiguous key data. Duplicate publications were excluded.

### Data extraction and quality assessment

Data from eligible studies were extracted using a standardized procedure and compiled into a comprehensive data table to ensure consistency and comparability across studies. The extracted information included: (1) basic study characteristics (author, publication year, region, and document type); (2) sampling characteristics (sampling time range, farm scale, swine herd type, clinical status, and sample type); (3) co-infection–related indicators (total samples, PEDV single infection count, PEDV single infection rate, genotype, co-infection positive cases, co-infection detection rate, co-infection spectrum); and (4) pathogen detection methods and NOS quality rating. To enhance the reliability and validity of the meta-analysis, a methodological quality assessment was performed using a predefined standardized procedure. A seven-item quality assessment checklist was developed based on the Newcastle-Ottawa Scale (NOS) and tailored to the epidemiological characteristics of this study. The checklist evaluated: (1) clarity of the sampling method; (2) traceability of sample sources; (3) adequacy of sample size (≥ 30); (4) control of potential confounding factors (e.g., age, immune and vaccination status, feeding and management conditions, and sampling season); (5) rationality and consistency of grouping; (6) reliability of pathogen detection methods; and (7) completeness of outcome indicators. Each item was scored as one point if the criterion was met, yielding a total score ranging from zero to seven. Studies were classified as low quality (zero -two points), medium quality (three-four points), or high quality (five-seven points).

### Risk assessment of publication bias and sensitivity analysis

The primary outcome of this study was the PEDV co-infection rate, and the effect size (ES) was defined as the pooled co-infection rate. Forest plots were used to present the pooled ES and its 95% confidence interval (CI). Given that proportion data approaching zero or one and small sample sizes are common in single-group rate meta-analyses, direct pooling of raw proportions may violate the normality assumption and introduce bias. Therefore, proportion data were transformed before pooling. The double arcsine transformation (DAT) was primarily applied to improve data normality, stabilize variance, and reduce the influence of extreme proportions on weighting and pooled estimates. Inter-study heterogeneity was assessed using Cochran’s Q test and the I² statistic. A fixed-effects model was used when heterogeneity was low (Q test *P* > 0.10 and I² ≤ 50%), whereas a random-effects model was applied in cases of moderate to high heterogeneity (*P* ≤ 0.10 or I² > 50%). Publication bias was evaluated visually using funnel plots and quantitatively using Egger’s and Begg’s tests. When potential publication bias was detected, the trim-and-fill method was applied to assess its impact on the pooled results. The robustness of the results was examined through sensitivity analyses by sequentially excluding individual studies. Stability was confirmed if the pooled ES remained consistent after exclusion of any single study; otherwise, the methodological quality and data consistency of the influential studies were further examined. In addition, subgroup analyses based on study quality were conducted to evaluate the influence of study quality on the overall conclusions.

### Subgroup analysis and meta-regression analysis

Based on the availability of PEDV-related raw data and its association with clinical and epidemiological characteristics, predefined subgroup analyses were conducted across multiple dimensions. All subgroups were strictly defined according to information reported in the original studies, using unified classification criteria and standardized data extraction procedures to ensure consistency and accuracy. Subgroup analyses were performed according to: (1) publication year (2010–2014, 2015–2020, and 2021–2025); (2) sampling region (China vs. other regions); (3) sampling time range (2010–2014, 2015–2019, 2020–2025, or not specified [NS]), determined by the sampling year for single-year studies or by the midpoint year for studies reporting a time interval; (4) farm scale (large-scale farms, backyard farms, or NS); (5) swine herd type (piglets, finishing pigs, mixed herds, or NS); (6) clinical status (diarrhea, mixed, or NS); (7) sample source (fecal, intestinal, mixed, other, or NS); (8) virus genotype (G1, G2, or NS); (9) detection method (RT-PCR, qRT-PCR, or other methods); (10) study quality based on the NOS assessment (high or medium quality); and (11) PEDV co-infection spectrum (dual, triple, or multiple infections [> three pathogens]). Differences in pooled co-infection rates among subgroups were assessed using tests for between-subgroup heterogeneity. When significant heterogeneity was detected, meta-regression analyses were performed to explore potential sources of variability. Given the substantial heterogeneity observed, inter-study variance was estimated using the restricted maximum likelihood (REML) method, with predefined subgroup variables included as covariates. Changes in heterogeneity were evaluated using tau² and I² statistics, and confidence intervals were adjusted using the Knapp–Hartung method to enhance robustness. A two-sided *P* < 0.05 was considered statistically significant.

### Spatiotemporal subgroup analysis of PEDV infection patterns in various regions of China

Based on the aforementioned overall subgroup analysis framework, this study further explored the regional distribution characteristics of PEDV co-infection and the temporal dynamics of the co-infection spectrum in China by conducting a China-specific subgroup analysis. Meanwhile, data on PEDV single infection rates were included for comparative analysis to systematically characterize the epidemiological features and core differences between single and co-infection spectrums. A two-dimensional spatiotemporal stratification strategy was adopted, with cross-stratification by geographical region and sampling period (2010–2014, 2015–2019, and 2020–2025). China was divided into five geographical regions: Northern China (Beijing, Tianjin, Hebei, Shanxi, Inner Mongolia, Liaoning, Jilin, and Heilongjiang); Eastern China (Shanghai, Jiangsu, Zhejiang, Anhui, Shandong, and Henan); Central–Southern China (Fujian, Jiangxi, Hunan, Guangdong, Guangxi, Hainan, and Taiwan); Southwestern China (Hubei, Chongqing, Sichuan, Guizhou, Yunnan, and Tibet); and Northwestern China (Shaanxi, Gansu, Qinghai, Ningxia, and Xinjiang). Based on these divisions, the pooled PEDV co-infection rate for each region was calculated to delineate regional prevalence levels and distributional differences in co-infection spectrums. For the temporal dimension, uniform four-five-year intervals were applied. PEDV single infection and co-infection rates in each region were estimated for each period. Through two-dimensional spatiotemporal comparison, temporal trends and regional evolutionary differences between the two infection patterns were clearly illustrated, providing data support for elucidating the spatiotemporal distribution of PEDV infections in China.

## Result

### Study selection and characteristics

**Fig. 1:**
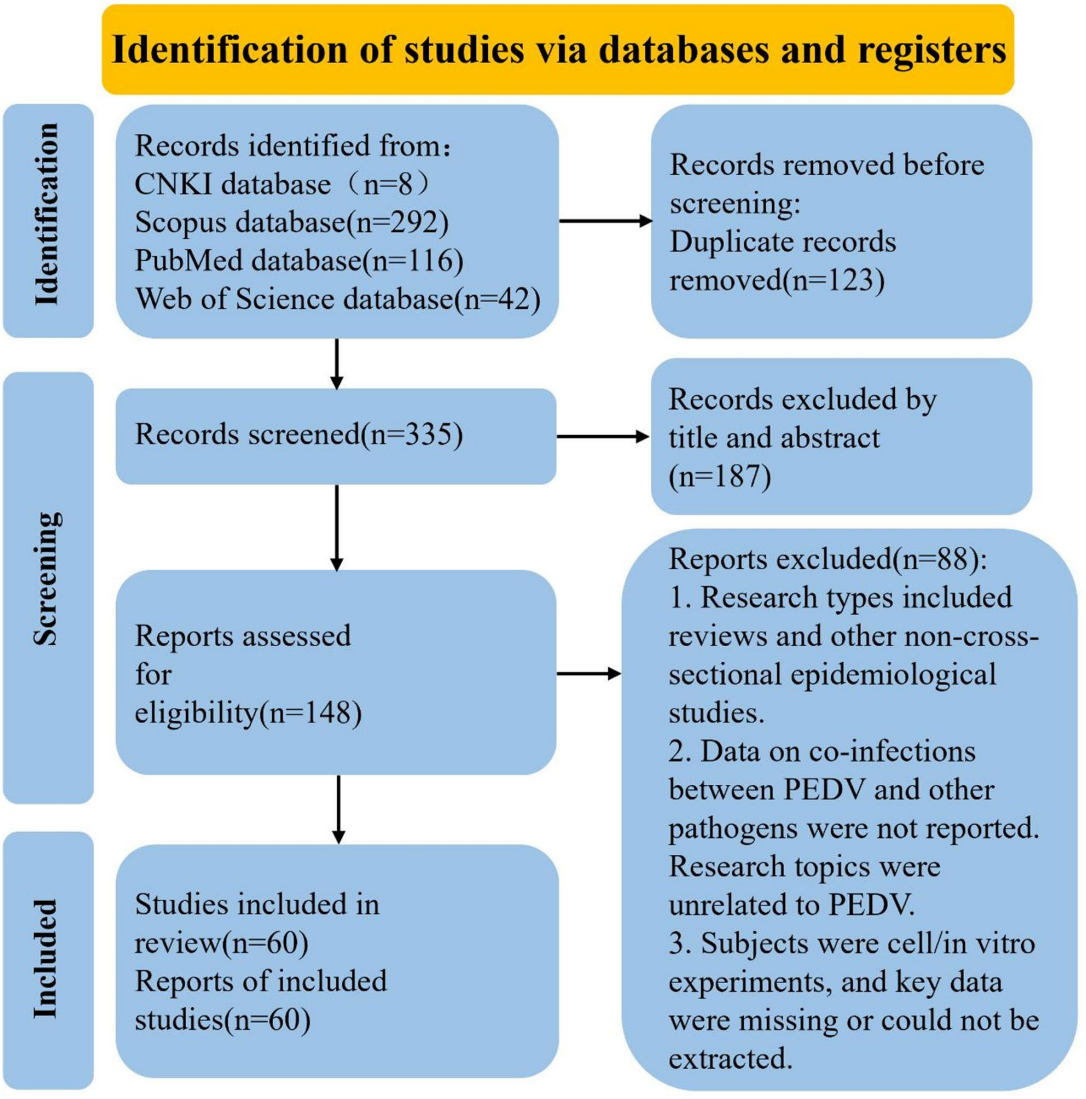
PRISMA flow diagram illustrating the study selection process for the systematic review and meta-analysis of porcine epidemic diarrhea virus (PEDV) co-infections. A total of 458 records were initially retrieved from four databases: CNKI (*n* = 8), PubMed (*n* = 116), Web of Science (*n* = 42), and Scopus (*n* = 292). Following the removal of 123 duplicate records, 335 studies were screened by title and abstract, with 187 excluded primarily for non-epidemiological study designs or irrelevance to PEDV co-infections. The remaining 148 studies were assessed via full-text review, and 88 were excluded due to incomplete key data or failure to report PEDV co-infection information. Finally, 60 eligible studies were included in the subsequent qualitative synthesis and quantitative meta-analysis

A systematic literature search was conducted across four databases, CNKI, Scopus, PubMed, and Web of Science, yielding a total of 458 potentially relevant records (eight from CNKI, 292 from Scopus, 116 from PubMed, and 42 from Web of Science). After deduplication using Zotero software, 335 records remained. Titles and abstracts were subsequently screened to assess eligibility according to predefined inclusion criteria. In total, 275 records were excluded for the following main reasons: non-cross-sectional epidemiological study designs (e.g., reviews), absence of data on PEDV co-infection with other pathogens, irrelevance to PEDV infection, studies limited to cell-based or in vitro experiments, and insufficient or non-extractable key data. Ultimately, 60 studies met the inclusion criteria, including 58 journal articles, one dissertation, and one conference paper, forming the complete dataset for subsequent quantitative analyses (Fig. 1). Table 1 summarizes the key characteristics of the 60 included studies.

**Table 1:** Basic characteristics of the 60 studies included in the systematic review and meta-analysis

Basic Study Characteristics					Sampling Characteristics				
Number	Author	Publication Year	Region	Document Type	Sampling Time Range	Farm scale	Swine Herd Type	Clinical Status	Sample Type
1	Zhao Zhenpeng et al. [18]	2015	China (Jiangsu, Jilin, Guangdong, Shanghai, Henan)	Academic Conference Paper	2012–2014	Large-scale	Piglets	Diarrhea	Fecal Samples
2	Zhang Letian [19]	2025	China (Henan, Jiangsu, Anhui, Guangdong)	Thesis	NS	Large-scale	Piglets	Diarrhea	Feces, anal swabs, digestive tract tissues
3	Jiao Wenqiang et al. [20]	2021	China (Henan, Hebei, Shandong, Shanxi, Jiangxi)	Journal Article	2014–2018	Large-scale	Piglets	Diarrhea	NS
4	Yan Zhao et al. [21]	2019	China (Hubei, Henan, Guangxi)	Journal Article	2017–2018	Large-scale	Finishing pigs	Diarrhea	Serum, lymph nodes, spleen, lungs, kidneys, intestinal tissues
5	Hongjin Zhou et al. [22]	2022	China (Guangxi)	Journal Article	2020–2022	Large-scale	Piglets	Diarrhea	Fecal Samples
6	Xin Huang et al. [23]	2019	China (Liaoning, Shandong, Chongqing, Shaanxi, Ningxia, Gansu, Henan)	Journal Article	2015–2018	Large-scale	Piglets	Diarrhea	Fecal Samples
7	Qianwen Wu et al. [24]	2024	China (Guangzhou)	Journal Article	NS	Large-scale	Piglets	Diarrhea	Fecal samples
8	Zhenhua Guo et al. [25]	2020	China (Henan)	Journal Article	2017	Large-scale	Piglets	Diarrhea	Intestinal tissue samples
9	Jiahui Guo et al.[10]	2024	China	Journal Article	2018–2021	Large-scale	Piglets	Diarrhea	Fecal samples, intestinal tissue samples
10	Jinghua Cheng et al. [26]	2022	China (Shanghai)	Journal Article	2017–2020	Large-scale	Piglets	Diarrhea	Fecal samples
11	Yaxiang Luo et al. [27]	2025	China (Guangxi)	Journal Article	NS	Large-scale	NS	Diarrhea	Fecal samples
12	Liu-Hui Zhang et al. [28]	2023	China (Henan and Shanxi)	Journal Article	2016–2021	Large-scale	Piglets	Diarrhea	Intestinal tissues, blood samples, fecal samples
13	M. Wang et al. [29]	2018	China (Gansu, Qinghai, and Sichuan)	Journal Article	2016–2017	Large-scale	NS	Diarrhea	Fecal samples
14	Haixin Huang et al. [30]	2020	China (Guangxi)	Journal Article	2017–2018	Large-scale	Finishing pigs	Diarrhea	Intestinal tissues, rectal swabs (diarrhea-related samples)
15	Qiaoling Zhang et al. [3]	2017	China (Gansu, Xinjiang, Henan, Hainan, Hunan, Shanxi)	Journal Article	2016–2017	Large-scale	NS	Diarrhea	Fecal samples
16	Yu Zhang et al. [31]	2019	China (18 provinces, including Anhui, Guangxi, Hubei, Jiangsu, etc.)	Journal Article	2016–2018	Large-scale	Sows, boars, piglets	Diarrhea	Feces, fecal swabs, and small intestinal tissues
17	Tien-Huan Hsu et al. [32]	2018	China (Taiwan Region)	Journal Article	2016–2017	Large-scale	Piglets	Diarrhea	Rectal swabs
18	Wei Wang et al. [6]	2025	China (multiple provinces, including Jiangsu, Shanghai, Anhui, Henan, Sichuan, Hunan, etc.)	Journal Article	2023–2025	Large-scale	Piglets	Diarrhea	Fecal samples
19	Francisco Jesús Castañeda Montes et al. [33]	2024	México (Aguascalientes, Guanajuato, Jalisco, Veracruz)	Journal Article	2019–2021	Backyard	Finishing pigs	NS	Serum samples
20	Zhonghao Xin et al. [34]	2024	China (Shandong)	Journal Article	2020–2023	Large-scale	NS	Diarrhea	Small intestinal tissues, intestinal contents
21	Jinzhu Zhou et al. [7]	2024	China (Jiangsu, Anhui, Zhejiang)	Journal Article	2018–2019	Large-scale	NS	Diarrhea	Serum samples
22	Lan-lan Zheng et al. [35]	2020	China (Henan)	Journal Article	NS	Backyard	Piglets	Diarrhea	Intestinal tissue samples
23	Yan Li et al. [36]	2023	China (pig farms in South China)	Journal Article	NS	Large-scale	NS	Diarrhea	Small intestinal tissue samples, anal swab samples
24	Jianpeng Chen et al. [37]	2023	China (Jiangsu, Shandong, Hubei, Guangdong, Hunan)	Journal Article	2022–2023	Large-scale	NS	Diarrhea	Fecal samples, small intestinal tissue samples
25	Jia-Wei Niu et al. [38]	2022	China (Guangdong)	Journal Article	2021–2022	Large-scale	NS	Diarrhea	Fecal samples, intestinal tissue samples
26	Hao-Ying Han et al. [39]	2019	China (Hebei and Henan)	Journal Article	2014–2018	Large-scale	Piglets	Diarrhea	Fecal samples, intestinal tissue samples
27	Jing Ren et al. [40]	2024	China (Shandong and Hebei)	Journal Article	NS	Large-scale	NS	Diarrhea	Fecal samples, rectal swab samples
28	Tingyu Luo et al. [41]	2024	China (Heilongjiang)	Journal Article	NS	Backyard	NS	With or without clinical diarrhea	Anal swab samples
29	Supatra Areekit et al. [42]	2022	Thailand (Bangkok and surrounding pig farms)	Journal Article	NS	Backyard	NS	Infected/symptomatic and non-infected groups	NS
30	Taveesak Janetanakit et al. [43]	2021	Thailand (73 pig farms in 20 provinces)	Journal Article	2014–2018	Large-scale	Piglets, breeders	Partially with diarrhea history, partially sub-clinically infected	Fecal samples, intestinal tissue samples
31	Chang Li et al. [44]	2022	China (Henan, Hubei, Jiangsu, Shandong, Guangdong, Hunan, Jiangxi, Sichuan)	Journal Article	2018–2021	NS	Piglets	Diarrhea	Intestinal samples, fecal samples
32	Yu Zang et al. [45]	2023	China (Shandong, Henan, Jiangsu, Fujian, Guangdong, Xinjiang)	Journal Article	2018–2022	Backyard	Diarrheic piglets (< 10 days old) and healthy pigs (> 1 month old)	Partially diarrheic, partially healthy	Intestinal samples
33	Zicheng Ma et al. [46]	2021	China (Shandong)	Journal Article	2015–2018	Large-scale	Sows, boars, piglets (< 100 days old), fattening pigs (≥ 100 days old)	NS	Tissue samples
34	Fanfan Zhang et al. [47]	2024	China (Guangdong, Guangxi, Jiangxi, Fujian, Hunan)	Journal Article	2021–2023	Large-scale	Sows, piglets, finishing pigs	Diarrhea	Small intestinal contents and tissue samples, fecal samples
35	Pei Zhu et al. [8]	2025	China (Yunnan)	Journal Article	2013–2022	Backyard	Piglets, finishing pigs, sows	Diarrhea	Fecal samples
36	Yue Zhang et al. [48]	2025	China (Henan, Hebei, Shanxi)	Journal Article	2020–2024	Backyard	Piglets	Diarrhea	Intestinal tissue samples
37	Caiwang Ye et al. [49]	2025	China (Fujian)	Journal Article	2016–2022	Large-scale	Piglets	Diarrhea	Intestinal tissue samples, fecal samples, and anal swab samples
38	Hechao Zhu et al. [50]	2024	China (Guangxi)	Journal Article	2022–2023	Backyard	Piglets	Diarrhea	NS
39	Jing Ren et al. [51]	2024	China (Gansu, Shanxi, Shaanxi, et al.)	Journal Article	2021–2022	Backyard	Finishing pigs	Diarrhea	Fecal samples, rectal swabs, oral fluid, oropharyngeal swabs
40	Jun Tu et al. [52]	2024	China (Guangxi)	Journal Article	2023–2024	Backyard	Finishing pigs	Diarrhea	Rectal swab samples, intestinal tissue samples
41	Wenwen Hou et al. [53]	2023	China (Jiangsu)	Journal Article	2021–2022	Backyard	Piglets	Diarrhea	Intestinal tissue samples
42	Yan Xiaoguang et al. [54]	2023	China (Henan, Shanxi, Hunan)	Journal Article	2018–2021	Backyard	Finishing pigs	Diarrhea	Intestinal tissue samples, fecal samples
43	Claudia Pérez-Rivera et al. [55]	2019	Mexico	Journal Article	2014–2017	Backyard	NS	NS	Rectal swab samples
44	Feng Wen et al. [56]	2021	China (Guangdong)	Journal Article	2018–2019	Backyard	Finishing pigs	Diarrhea	Fecal samples
45	Zhi-Li Li et al. [57]	2012	China (Guangdong, Jiangxi, Guangxi, Sichuan, Fujian, Jiangsu)	Journal Article	2010–2011	Backyard	Piglets	Diarrhea	Fecal samples
46	Montserrat-Elemi García-Hernández et al. [58]	2021	Mexico	Journal Article	NS	Large-scale	NS	Diarrhea	Rectal swab samples, intestinal tissue samples
47	Chen Wang et al. [59]	2016	China (Gansu)	Journal Article	NS	Backyard	Piglets, finishing pigs	All piglets have diarrhea	Fecal samples
48	D. Song et al. [60]	2015	China (Jiangxi)	Journal Article	2012–2015	Backyard	Sows, piglets	Diarrhea	Fecal samples, intestinal samples
49	Qian Zhang et al. [61]	2013	China	Journal Article	2011–2012	Backyard	Pigs of different age groups	Diarrhea; healthy	Fecal samples, intestinal samples
50	Wang Yidan et al. [62]	2023	China (Sichuan)	Journal Article	2020–2021	Backyard	Finishing pigs	Diarrhea	Fecal sample
51	Chunyan Jiang et al. [63]	2019	China (Zhejiang, Jiangsu, Fujian and Shanghai)	Journal Article	2013–2017	Backyard	Piglets	Diarrhea	Fecal sample
52	Fang Wu et al. [64]	2024	China (Sichuan)	Journal Article	2023–2024	Backyard	Piglets	Diarrhea	Small intestinal samples
53	Yu Feng et al. [65]	2020	China (Sichuan)	Journal Article	2017–2019	Backyard	Piglets, sows	Diarrhea	Fecal samples, intestinal samples
54	Fanfan Zhang et al. [66]	2019	China (Jiangxi, Zhejiang, Fujian, Guangdong, Hunan)	Journal Article	2012–2018	Backyard	Sows, piglets, finishing pigs	Diarrhea	Intestinal samples, fecal samples, milk samples
55	G. Jang et al. [67]	2017	South Korea	Journal Article	2014–2016	Large-scale	Piglets	Diarrhea	Small intestinal samples, fecal samples
56	Honglei Zhang et al. [68]	2019	China (Henan)	Journal Article	2015–2018	Backyard	Piglets, finishing pigs, sows	Diarrhea	Fecal samples, intestinal samples
57	Ling Zhou et al. [69]	2019	China (Guangdong)	Journal Article	2016–2017	Backyard	Piglets, sows	Diarrhea	Fecal samples, intestinal content samples
58	Hong-Xuan Li et al. [70]	2023	China (Henan)	Journal Article	2019–2021	Large-scale	Piglets	Diarrhea	Fecal samples, intestinal tissue samples
59	Ying Shi et al. [71]	2021	China (Shanghai)	Journal Article	2015–2018	Backyard	Piglets	Diarrhea	Fecal samples
60	Guangbin Si et al. [72]	2021	China (Guangdong, Guangxi, Jiangxi, Fujian)	Journal Article	2016–2018	Large-scale	Finishing pigs	Diarrhea	Intestinal samples

	Co-infection–related Indicators							Pathogen Detection Methods	NOS Quality Rating
Number	Total Samples	PEDV Single Infection Count	PEDV Single Infection Rate (%)	Genotype	Co-infection Positive Cases	Coinfection Detection Rate (%)	Co-infection spectrum		
1	314	76	24.20%	G1	47	14.97%	PEDV and PKV	RT-PCR	high
2	104	11	10.58%	G1/G2	10	9.62%	PEDV and PoRV	qRT-PCR	high
3	191	178	93.19%	NS	13	6.81%	PEDV and PDCoV	RT-PCR	high
4	117	16	13.68%	G1	1	0.85%	PEDV and PRRSV	RT-PCR	high
5	3236	543	16.78%	NS	48	1.48%	PEDV and TGEV; PEDV and PDCoV	qRT-PCR	high
6	245	11	4.49%	NS	20	8.16%	PEDV and PDCoV; PEDV and TGEV; PEDV, PDCoV, and TGEV	qRT-PCR	high
7	64	9	14.06%	NS	3	4.69%	PEDV and PoRV	Dual ERA	high
8	76	5	6.58%	NS	48	63.16%	PEDV and PCV	RT-PCR	high
9	4468	NS	NS	NS	120	2.69%	PEDV and PDCoV; PEDV and TGEV	Genomic sequencing analysis	medium
10	1078	NS	NS	NS	185	17.16%	PEDV and BVDV	qRT-PCR	high
11	30	12	40.00%	NS	4	13.33%	PEDV and PoRV	RT-PCR	high
12	175	14	8.00%	NS	12	6.86%	PEDV and PCLV	RT-PCR	high
13	189	49	25.93%	NS	3	1.59%	PEDV and PDCoV	RT-PCR	high
14	103	NS	NS	NS	15	14.56%	PEDV and PoBuV	qRT-PCR	medium
15	116	48	41.38%	G2	13	11.21%	PEDV and PKV	qRT-PCR	high
16	719	233	32.41%	NS	34	4.73%	PEDV and PDCoV	RT-PCR	high
17	172	22	12.79%	NS	16	9.30%	PEDV and PDCoV; PEDV and TGEV; PEDV, PDCoV and TGEV	qRT-PCR	high
18	348	35	10.06%	NS	117	33.62%	PEDV and PDCoV; PEDV and PoRVA; PEDV, PDCoV and PoRVA	qRT-PCR	high
19	516	163	31.59%	NS	165	31.98%	PEDV and PDCoV	ELISA	high
20	97	14	14.43%	NS	20	20.62%	PEDV and PoRVA; PEDV and TGEV; PEDV, TGEV and PoRVA	qRT-PCR	high
21	121	45	37.19%	NS	8	6.61%	PEDV and TGEV	RT-PCR	high
22	63	36	57.14%	NS	18	28.57%	PEDV and PBoV	qRT-PCR	high
23	160	56	35.00%	NS	5	3.13%	PEDV and TGEV; PEDV and PDCoV; PEDV, TGEV, and PDCoV	qRT-PCR	medium
24	462	91	19.70%	NS	177	38.31%	PEDV and TGEV; PEDV and PDCoV; PEDV, TGEV, and PDCoV	qRT-PCR	high
25	94	15	15.96%	NS	6	6.38%	PEDV and PDCoV; PEDV and TGEV; PEDV, TGEV, and PDCoV	RT-PCR	high
26	66	29	43.94%	NS	18	27.27%	PEDV and PCV	qRT-PCR	high
27	356	112	31.46%	NS	232	65.17%	PEDV and Bhyo; PEDV and LI; PEDV, Bhyo, and LI	qRT-PCR	medium
28	256	0	0.00%	NS	7	2.73%	PEDV and PoRV	qRT-PCR	high
29	50	16	32.00%	NS	14	28.00%	PEDV and PCV	LAMP-LFD	medium
30	777	NS	NS	NS	236	30.37%	PEDV and EV-G	RT-PCR	medium
31	7107	NS	NS	G2	123	1.73%	PEDV and PoRV; PEDV and PDCoV; PEDV and TGEV; PEDV, PoRV and PDCoV	RT-PCR	medium
32	324	13	4.01%	NS	19	5.86%	PEDV and PKV	qRT-PCR	high
33	1325	NS	NS	NS	17	1.28%	PEDV and PCV	RT-PCR	high
34	1971	696	35.31%	G2	199	10.10%	PEDV and PoRV; PEDV and PDCoV; PEDV, PDCoV and PoRV; PEDV, TGEV and PoRV; PEDV, SADS-CoV and PoRV	qRT-PCR	high
35	1851	427	23.07%	G2	89	4.81%	PEDV and PSaV; PEDV and PoRV; PEDV, PSaV and PaStV	RT-PCR	high
36	148	23	15.54%	NS	12	8.11%	PEDV and PDCoV	cdPCR	high
37	231	105	45.45%	NS	5	2.16%	PEDV and PEAV; PEDV and PDCoV; PEDV and TGEV	qRT-PCR	high
38	5483	468	8.54%	NS	30	0.55%	PEDV and PoRVA; PEDV and PDCoV; PEDV and TGEV; TGEV, PEDV, and PoRVA	qRT-PCR	high
39	4800	315	6.56%	NS	644	13.42%	PEDV and PoRV; PEDV and PDCoV; PEDV, PDCoV and PoRV	qRT-PCR	high
40	1502	66	4.39%	NS	198	13.18%	PEDV and PKV; PEDV and TGEV; PEDV, TGEV, and PKV	qRT-PCR	high
41	112	32	28.57%	G2	26	23.21%	PEDV and PoRV	qRT-PCR	high
42	48	8	16.67%	NS	3	6.25%	PEDV and PDCoV	RT-PCR	high
43	885	NS	NS	NS	62	7.01%	PEDV and PDCoV; PEDV, PDCoV, and TGEV	qRT-PCR	high
44	34	16	47.06%	G2	2	5.88%	PEDV and RV	RT-PCR	high
45	127	40	31.50%	NS	15	11.81%	PEDV and TGEV	RT-PCR	high
46	74	23	31.08%	NS	28	37.84%	PEDV and PKV; PEDV, PKV and PSaV	RT-PCR	medium
47	203	18	8.87%	NS	153	75.37%	PEDV and PKV; PEDV and SaV; PEDV, PKV, and SaV	RT-PCR	high
48	356	161	45.22%	NS	70	19.66%	PEDV and PDCoV	RT-PCR	high
49	1212	137	11.30%	NS	603	49.75%	PEDV and PKoV; PEDV and PBoV; PEDV, PKoV, and PBoV; PEDV, PKoV, PBoV and GARV	qRT-PCR	high
50	242	5	2.07%	NS	25	10.33%	PEDV and GARV; PEDV and PDCoV; PEDV, GARV and PDCoV	qRT-PCR	high
51	402	187	46.52%	NS	43	10.70%	PEDV and PSV; PEDV and SaV; PEDV, PSV, and SaV	RT-PCR	high
52	352	80	22.73%	NS	97	27.56%	PEDV and PCV	qRT-PCR	high
53	634	157	24.76%	NS	47	7.41%	PEDV and PDCoV	RT-PCR	high
54	2987	1360	45.53%	G2	405	13.56%	PEDV and PDCoV; PEDV and TGEV; PEDV and PoRV; PEDV and SADS-CoV; PEDV, PDCoV and PoRV; PEDV, PDCoV and TGEV	RT-PCR	high
55	683	NS	NS	NS	45	6.59%	PEDV and PDCoV; PEDV, PDCoV, and PRV	RT-PCR	medium
56	430	NS	NS	NS	101	23.49%	PEDV and PDCoV	RT-PCR	medium
57	170	63	37.06%	NS	66	38.82%	PEDV and SADS-CoV; PEDV and RV; PEDV and PDCoV; PEDV, SADS-CoV and RV; PEDV, SADS-CoV, RV and PDCoV	RT-PCR	high
58	65	4	6.15%	G2	22	33.85%	PEDV and PCV	qRT-PCR	high
59	753	46	6.11%	NS	42	5.58%	PEDV and PKoV; PEDV and PAstV; PEDV and PSV	Luminex xTAG	high
60	181	26	14.36%	NS	37	20.44%	PEDV and PDCoV; PEDV and SADS-CoV; PEDV and PRV-A	RT-PCR	high

NS: Not specified (information was not provided in the original study)

### Results of publication bias and sensitivity analysis

**Fig. 2:**
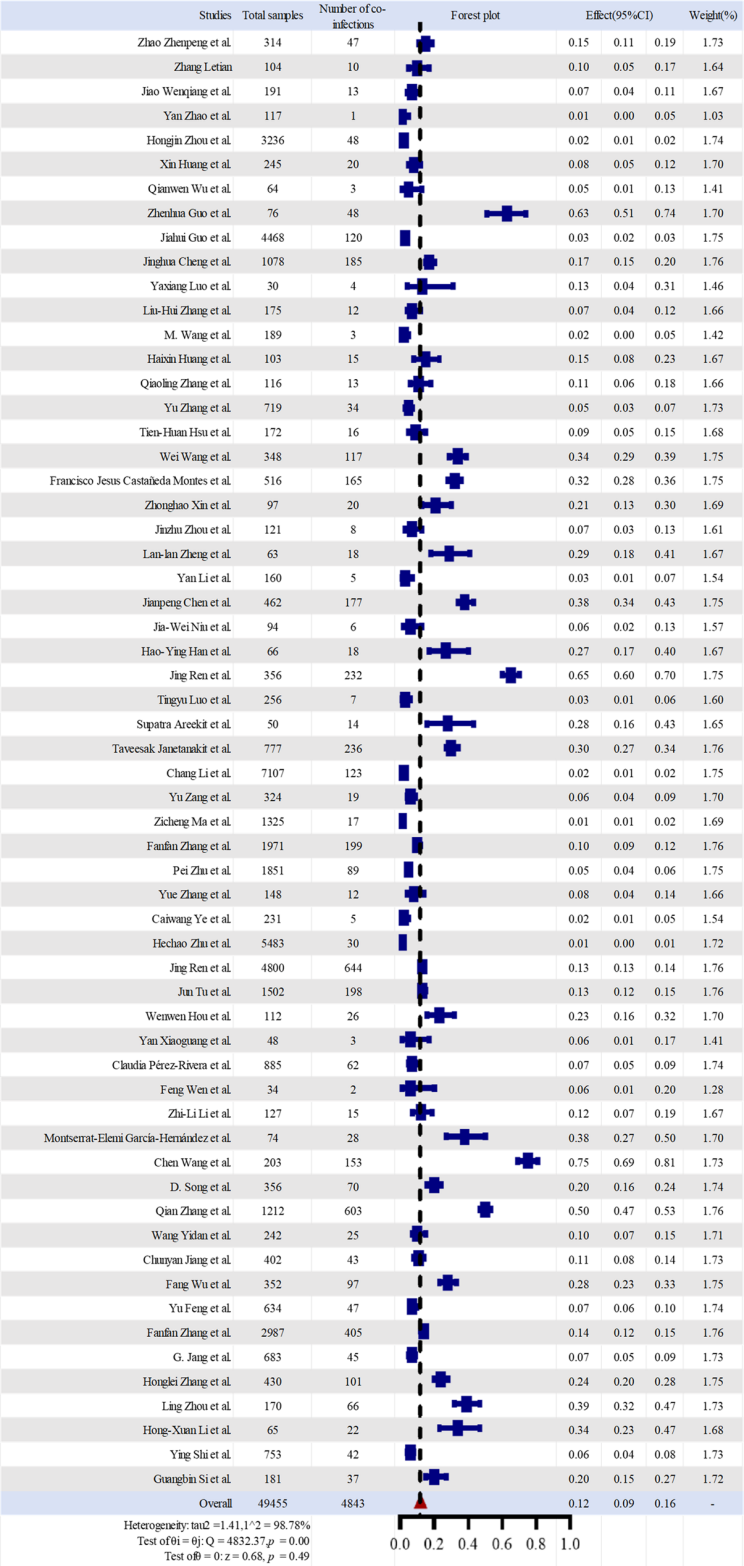
Forest plot of PEDV co-infection with other pathogens using a random-effects model. The left panel lists the included studies (first author), total sample size, and number of PEDV co-infection positive cases. In the forest plot, squares represent individual study estimates, horizontal lines indicate 95% CIs, the filled triangle and the vertical dashed line represent the pooled co-infection rate. Substantial heterogeneity was observed among studies (I²= 98.78%, *P* < 0.001). The right panel presents the effect estimates (95% CI) and the corresponding study weights contributing to the pooled result

A total of 60 studies comprising 49,455 samples were included in the analysis, among which 4,843 samples were positive for PEDV co-infection. Substantial between-study heterogeneity was observed (I² = 98.78%, *P* < 0.001); therefore, a random-effects model was applied using Stata MP 17 software. A forest plot was generated to display the pooled effect estimate and its 95% confidence interval (CI) (Fig. 2). The pooled co-infection rate of PEDV with other pathogens was 0.12 (95% CI: 0.09–0.16), corresponding to an overall prevalence of 12%. In total, 121 distinct PEDV co-infection spectrums were identified. Dual infections accounted for the largest proportion (83.47%), followed by triple infections (14.88%), while quadruple infections were least frequent (1.65%). Among dual infections, PEDV-PDCoV was the most common combination (23.1%), followed by PEDV-TGEV (13.2%), PEDV-PoRV (10.7%), PEDV-PKV (5.7%), and PEDV-PCV (4.1%). PEDV-SaV, PEDV-PKoV, and PEDV-SADS-CoV each accounted for 2.4% of dual co-infections.

Visual inspection of the funnel plot indicated that effect sizes were approximately symmetrically distributed around the pooled estimate, with only slight asymmetry observed among small-sample studies. Publication bias was further assessed using Egger’s regression test and Begg’s rank correlation test. The results showed no statistically significant publication bias (Egger’s test: t = − 1.295, *P* = 0.200; Begg’s test: z = − 0.727, *P* = 0.467). Taken together with the funnel plot, these findings suggest that publication bias had a limited impact on the pooled estimates.

Sensitivity analyses demonstrated good robustness of the pooled effect size. Sequential exclusion of individual studies did not materially alter the pooled PEDV co-infection rate, which remained stable at approximately 0.12 (95% CI: 0.09–0.18), indicating that no single study unduly influenced the overall results. Stratified sensitivity analysis based on NOS scores revealed a clear association between study quality and effect estimation. After exclusion of 10 medium-quality studies, the pooled co-infection rate increased to 0.16 (95% CI: 0.12–0.19), accompanied by a narrower 95% CI and improved precision. In contrast, exclusion of the 50 high-quality studies resulted in a further increase in the pooled estimate to 0.20 (95% CI: 0.10–0.31), with a markedly wider 95% CI and reduced estimation precision.

### Results of subgroup analysis

**Fig. 3:**
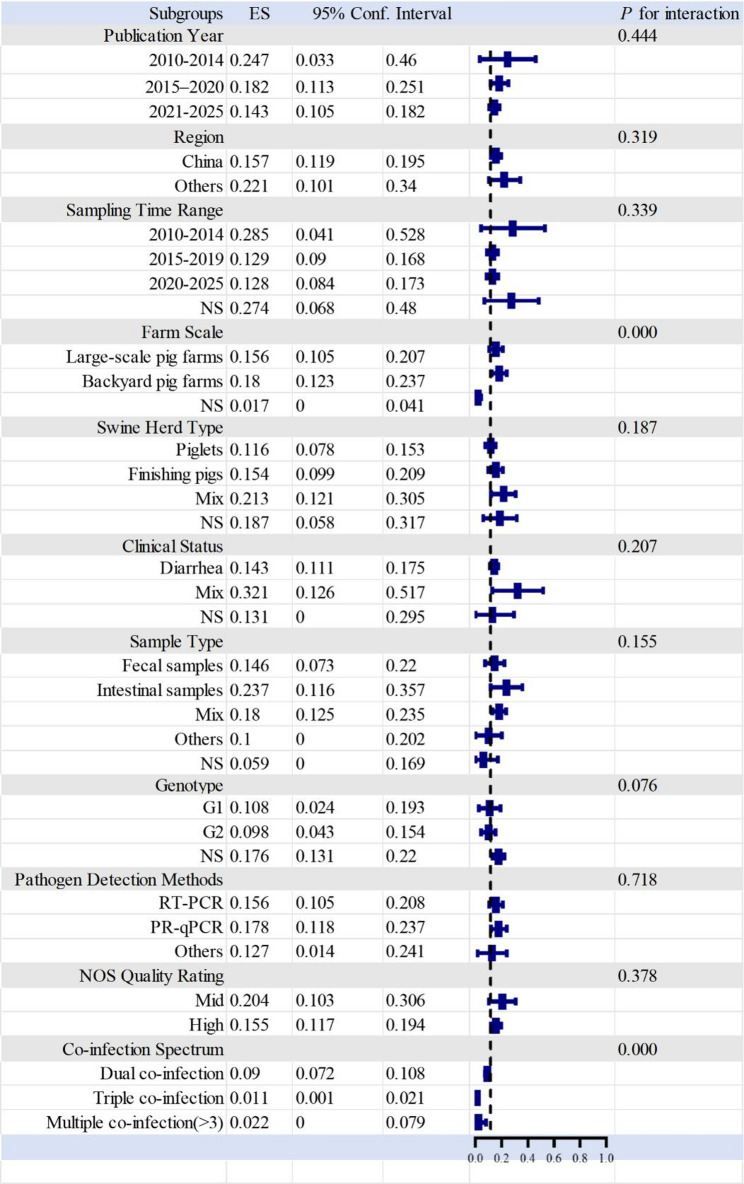
Forest plot of subgroup analyses for PEDV co-infection. Subgroup analyses were conducted to assess the influence of different study characteristics on the pooled co-infection rate of porcine epidemic diarrhea virus (PEDV) and to explore potential sources of between-study heterogeneity. Subgroups represent the categories of stratified variables; ES denotes the pooled co-infection rate for each subgroup, with corresponding 95% confidence intervals (CIs). P for interaction indicates the statistical significance of differences in pooled effect sizes between subgroups. Significant inter-subgroup differences were observed for stratification by farm scale and PEDV co-infection type (both *P* < 0.001), suggesting that these factors are major contributors to inter-study heterogeneity

To investigate potential sources of between-study heterogeneity, subgroup analyses were conducted according to 11 predefined study characteristics to assess their influence on the pooled PEDV co-infection rate. Inter-subgroup interaction tests indicated that only two factors, farm scale and PEDV co-infection type, were associated with statistically significant differences in pooled effect sizes (both *P* < 0.001), identifying them as the primary contributors to heterogeneity. No significant inter-subgroup differences were observed for the remaining nine stratification factors, including publication year, sampling region, sampling time, swine herd type, clinical status, sample type, virus genotype, detection method, and NOS quality score (all *P* > 0.05), suggesting that these variables did not substantially explain the observed heterogeneity (Fig. 3). To delineate the sources of heterogeneity further, meta-regression analyses were performed within subgroups defined by the two key factors: farm scale and PEDV co-infection type. High residual heterogeneity persisted in large-scale farms (I² = 89.50%), smallholder farms (I² = 93.80%), and dual co-infection groups (I² = 83.70%). The pooled PEDV co-infection rate was highest in smallholder farms (ES = 0.180, 95% CI: 0.127–0.237), followed by farms with large-scale (ES = 0.156, 95% CI: 0.105–0.207), and lowest in unspecified scale farms (ES = 0.017, 95% CI: 0-0.041). Owing to the limited number of studies reporting farms with unspecified scale, this subgroup was excluded from subsequent meta-regression analyses. Decomposition of within-subgroup heterogeneity showed that, in the large-scale farm subgroup, sampling region and swine herd clinical status contributed to heterogeneity but with relatively low impact (I²< 50%) (Table 2). In contrast, NOS quality score and co-infection spectrum were the dominant sources, accounting for 67.1% and 57.61% of the heterogeneity, respectively. In the smallholder farm subgroup, the extremely high heterogeneity was primarily attributable to the co-infection spectrum (I²= 55.13%), whereas the contributions of most other factors were minimal or negligible. Stratified analysis by co-infection type revealed that dual infections had the highest pooled co-infection rate (ES = 0.090, 95% CI: 0.072–0.108), followed by multiple infections involving more than three pathogens (ES = 0.022, 95% CI: 0.000-0.079), while triple infections exhibited the lowest rate (ES = 0.011, 95% CI: 0.000-0.001). Meta-regression within the dual co-infection subgroup indicated multiple moderate sources of heterogeneity, including sampling region (I²= 62.34%), swine herd clinical status (I = 59.11%), and NOS quality score (I²= 57.49%). Additional contributions were observed for farm scale (I²= 28.37%), swine herd type (I²= 39.78%), and pathogen detection method (I²= 36.76%).

**Table 2:** Results of meta-regression analysis conducted within subgroups stratified by farm scale and PEDV co-infection type

Subgroup	tau²	I²	*P*
**Large-scale**	0.02	89.50%	0.000
Publication year	0.00	0.00%	0.000
Region	0.30	18.69%	0.000
Sampling time range	0.00	0.00%	0.000
Swine herd type	0.01	0.00%	0.000
Clinical status	0.21	14.53%	0.000
Sample type	0.00	0.00%	0.000
Genotype	0.00	0.00%	0.000
Pathogen detection methods	0.22	0.00%	0.000
NOS quality rating	0.20	67.10%	0.000
Co-infection spectrum	1.62	57.61%	0.000
**Backyard**	0.02	93.80%	0.000
Publication year	0.00	0.00%	0.000
Region	0.07	0.00%	0.000
Sampling time range	0.00	0.00%	0.000
Swine herd type	0.18	0.00%	0.000
Clinical status	0.00	0.00%	0.000
Sample type	0.00	0.00%	0.000
Genotype	0.00	0.00%	0.000
Pathogen detection methods	0.00	0.00%	0.000
NOS quality rating	0.07	1.76%	0.000
Co-infection spectrum	1.55	55.13%	0.000
**Dual co-infection**	0.01	83.70%	0.000
Publication year	0.00	0.00%	0.000
Region	1.76	62.34%	0.000
Sampling time range	0.00	0.00%	0.000
Farm scale	0.76	28.37%	0.000
Swine herd type	1.89	39.78%	0.000
Clinical status	1.69	59.11%	0.000
Sample type	0.48	0.00%	0.000
Genotype	0.00	0.00%	0.000
Pathogen detection methods	1.18	36.76%	0.000
NOS quality rating	1.56	57.49%	0.000

### Spatio-temporal characteristics of PEDV infection patterns in different regions of China

**Fig. 4:**
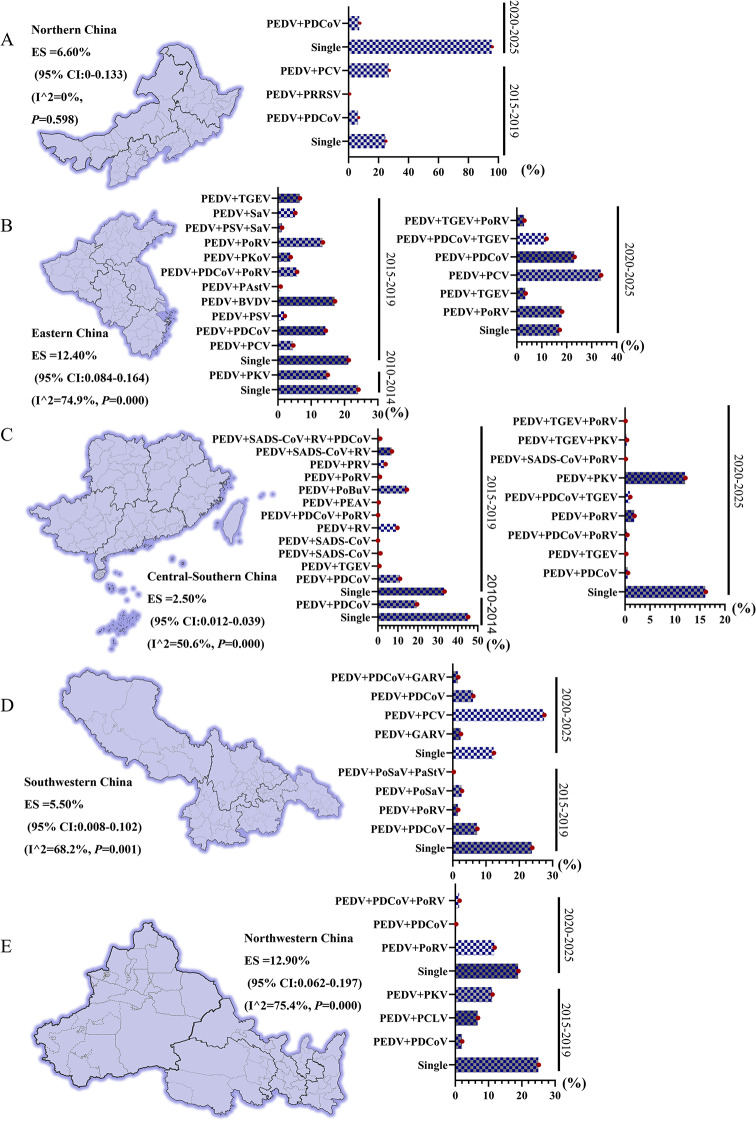
Spatio-temporal characteristics of PEDV infection patterns across five major geographical regions of China. **A**: Northern China (2015–2025) is dominated by single PEDV infections; co-infections are mainly PEDV-PCV and PEDV-PDCoV, with an overall co-infection rate of 6.6% (*P* = 0.590), showing no significant difference compared with other regions. **B**: Eastern China (2010–2025) exhibits the most complex infection patterns, including diverse dual and triple co-infections, with an overall co-infection rate of 12.4% (*P* < 0.001), significantly differing from other regions; the PEDV single infection rate remains relatively stable at approximately 22%. **C**: Central-Southern China (2010–2025) is characterized by frequent multiple co-infections involving PEDV and other pathogens, while the overall co-infection rate is low (2.50%, *P* < 0.05); the PEDV single infection rate remains relatively stable at approximately 25%. **D**: Southwestern China (2015–2025) is dominated by single PEDV infections (19%); co-infections are primarily dual infections involving PEDV-PCV and PEDV-PDCoV, with an overall co-infection rate of 5.50% (*P* < 0.05). **E**: Northwestern China (2015–2025) is also dominated by single PEDV infections (23%); co-infections mainly involve PEDV-PoRV and PEDV-PKV, with an overall co-infection rate of 12.9% (*P* < 0.001)

The 60 included studies were stratified by five geographical regions of China and three time periods (2010–2014, 2015–2019, and 2020–2025) to examine regional and temporal patterns of PEDV co-infection (Fig. 4). In the northern region, six studies contributed six effect sizes. The pooled co-infection rate of PEDV with other pathogens was 6.60% (95% CI: 0-0.133). Heterogeneity was negligible (I²= 0%, *P* = 0.598), indicating no significant variability within or between regions. Temporal analysis showed a marked increase in the single PEDV infection rate from 29.12% in 2015–2019 to 93% in 2020–2025, while the corresponding co-infection rates remained relatively stable (8.56% and 7.65%, respectively). During 2015–2019, co-infections involved multiple pathogens (e.g., PRRSV, PCV, PDCoV), whereas in 2020–2025, co-infections were predominantly associated with PDCoV, accompanied by an increased proportion of single PEDV infections and a reduced diversity of co-infecting pathogens.

In the eastern region, fifteen studies contributed 28 effect sizes. The pooled PEDV co-infection rate was 12.40% (95% CI: 0.084–0.164), with substantial heterogeneity (I²= 74.9%, *P* < 0.001). The single PEDV infection rate showed a gradual decline over time, decreasing from 25% in 2010–2014 to 22% in 2015–2019 and 19% in 2020–2025. The co-infection rate decreased from 19% in 2010–2014 to 7.44% in 2015–2019, followed by an increase to 13.21% in 2020–2025. Co-infection spectrums were relatively simple during 2010–2014, primarily involving PCV. Between 2015 and 2019, pathogen diversity increased markedly, with involvement of nine pathogens (including PCV, PDCoV, and BVDV) and multiple co-infection combinations. During 2020–2025, pathogen combinations became more concentrated, dominated by PoRV, TGEV, PCV, PDCoV, and their combinations.

In the central-southern region, seventeen studies contributed 47 effect sizes. The pooled PEDV co-infection rate was 2.50% (95% CI: 0.012–0.039), with moderate heterogeneity (I²= 50.6%, *P* < 0.001). A continuous decline in the single PEDV infection rate was observed, from 47% in 2010–2014 to 36% in 2015–2019 and 16% in 2020–2025. The co-infection rate showed a parallel decrease, declining from 19.66% to 2.70% and further to 1.14% across the same periods. Despite the overall reduction, co-infection spectrums exhibited considerable diversity. During 2010–2014, co-infections were mainly limited to PDCoV, with no multiple co-infection events. In 2015–2019, pathogen diversity increased substantially, involving PDCoV, TGEV, and SADS‑CoV, including a quadruple infection (SADS‑CoV-RV-PDCoV) with a rate of 1.18%. In 2020–2025, co-infection spectrums became more complex, with frequent dual infections involving PDCoV, TGEV, PoRV, and PKV, as well as multiple co-infection combinations.

In the southwestern region, four studies contributed nine effect sizes. The pooled PEDV co-infection rate was 5.50% (95% CI: 0.008–0.109), with moderate heterogeneity (I²= 68.2%, *P* < 0.01). The single PEDV infection rate decreased from 25% in 2015–2019 to 17% in 2020–2025. The co-infection rate increased from 2.20% in 2015–2019, mainly involving PSaV, PoRV, PDCoV, and PSaV-PaStV, to 11.31% in 2020–2025, predominantly involving PDCoV, GARV, PDCoV-GARV, and PCV.

In the northwestern region, five studies contributed 13 effect sizes. The pooled PEDV co-infection rate was 12.90% (95% CI: 0.062–0.197), with substantial heterogeneity (I²= 75.4%, *P* < 0.001). The single PEDV infection rate declined from 25% in 2015–2019 to 21% in 2020–2025. The co-infection rate decreased from 6.44% to 4.47% over the same periods, with dominant pathogen combinations shifting from PCLV, PSaV, PKV, and PDCoV to PoRV, PDCoV, and PDCoV-PoRV.

## Discussion

In summary, this study systematically evaluated PEDV co-infection spectrums through a meta-analysis of 60 studies encompassing 49,455 samples worldwide. The pooled co-infection rate of PEDV with other pathogens was estimated at 12% (95% CI: 0.09–0.16), indicating that PEDV co-infections have transitioned from sporadic concurrent events to a recurrent epidemiological pattern coexisting with single PEDV infections within the swine production system.

Subgroup analyses identified farm size and co-infection type as the principal contributors to heterogeneity in PEDV co-infection rates (both *P* < 0.001), whereas the remaining nine stratified factors showed no significant regulatory effects. Further stratification revealed distinct heterogeneity structures across subgroups. In large-scale farms, heterogeneity was mainly attributable to study quality (NOS score, 67.1%) and infection patterns (57.61%), while sampling region and clinical status contributed to a lesser extent. In family farms, heterogeneity was predominantly driven by co-infection spectrums (I²= 55.13%), with minimal contributions from other factors. In the co-infection type subgroup, heterogeneity arose from multiple moderate contributors, including sampling region (62.34%), clinical status (59.11%), and study quality (57.49%), with no single dominant source identified. Notably, the co-infection rate in family farms (ES = 0.180, 95% CI: 0.127–0.237) was significantly higher than that in large-scale farms (ES = 0.156, 95% CI: 0.105–0.207), suggesting that differences in biosecurity levels may play an important role. Double infections represented the predominant co-infection spectrum, accounting for 83% of cases (ES = 0.090, 95% CI: 0.072–0.108), whereas triple (ES = 0.011) and higher-order infections (ES = 0.022) occurred at much lower frequencies. This distribution may reflect biological constraints on pathogen coexistence. PEDV infection may compromise the intestinal mucosal barrier, thereby facilitating secondary invasion by other enteric pathogens such as PDCoV or PCV, or conversely, prior infection with these pathogens may increase host susceptibility to PEDV [10]. In contrast, higher-order co-infections likely require the simultaneous breach of multiple immune barriers and may be limited by pathogen competition for host resources [73]. Nevertheless, the interactions among PEDV and co-infecting pathogens remain incompletely understood and warrant further experimental investigation to clarify their effects on viral pathogenicity and disease severity. Notably, the assessment of the co‑infection spectrum is subject to a high risk of bias, as most original studies targeted only a restricted set of pathogen combinations. This methodological limitation likely favored the detection of dual infections over more complex triple or quadruple co‑infections. Consequently, the adoption of broad‑spectrum pathogen detection methods is warranted in future epidemiological studies.

To further elucidate epidemic characteristics, spatiotemporal patterns of PEDV infection were analyzed across China’s five major geographical regions. Pronounced regional heterogeneity was observed. The northern region exhibited a low and stable co-infection rate (6.60%, I² = 0%), alongside a marked increase in single PEDV infections from 29.12% in 2015–2019 to 93% in 2020–2025, with co-infections largely restricted to PDCoV. The eastern region showed the highest co-infection rate (12.40%, I² = 74.9%) and the most complex pathogen combinations, despite a gradual decline in single infection rates. The central–southern region displayed the lowest co-infection rate (2.50%, I² = 50.6%), with concurrent declines in both single and co-infection rates over time, while maintaining diverse co-infection spectrums. The southwestern region demonstrated an opposite trend, characterized by decreasing single infections and increasing co-infections, accompanied by the continual emergence of novel pathogen combinations. In the northwestern region, co-infection rates remained relatively high (12.90%, I² = 75.4%), with stable dominance of PoRV-PDCoV combinations. In northern regions, we observed a marked increase in the single-infection rate of PEDV, raising the question of whether this trend is associated with the emergence and potential replacement by highly pathogenic G2 strains. Previous studies have proposed that these G2 variants may occupy the host ecological niche through rapid replication, thereby limiting co-infection with other pathogens [74]. However, our current dataset lacks definitive genotypic evidence to substantiate this hypothesis. Specifically, we were unable to confirm whether G2 variants are uniquely distributed in northern regions, nor could we exclude the possibility that these variants are also present in other regions without exerting comparable epidemiological effects. Moreover, the molecular mechanisms underlying such putative competitive exclusion remain poorly understood and warrant targeted experimental investigation. It is also plausible that this observed pattern results from the combined influence of multiple factors, including region-specific improvements in biosecurity practices, environmental heterogeneity, frequent animal movement, changes in vaccination strategies that impose distinct selective pressures on co-circulating pathogens, and the evolving landscape of population immunity [75]. Taken together, these findings suggest that PEDV prevention and control strategies may benefit from region-specific optimization to better align with local epidemiological characteristics and production systems. Future molecular epidemiological studies integrating viral genetic data with comprehensive field surveillance are urgently needed to elucidate the true drivers of these spatiotemporal distribution patterns.

Despite the systematic analysis of PEDV coinfection characteristics in this study, several limitations should be acknowledged. First, literature retrieval was restricted to four major databases, which may have resulted in the omission of relevant studies from other sources. Although a comprehensive search strategy was employed, variations in database indexing and search syntax may have led to the omission of some relevant studies. In addition, studies published in languages other than those searched may not have been captured, thereby limiting the global generalizability of our findings. Second, incomplete reporting in the included studies limited the depth of subgroup analyses; some subgroups contained relatively few studies, and key variables such as sampling time, farm size, and clinical status were not consistently specified. Uneven study distribution across subgroups, particularly for triple or higher-order infections, may have introduced bias. Third, although farm size and co-infection type were identified as major sources of heterogeneity, unmeasured confounding factors—including regional climate, vaccination coverage, and antimicrobial usage—could not be fully adjusted for. In addition, most included studies were conducted in China, with relatively few international reports, limiting global generalizability. Finally, the lack of individual-level data on immune status and farm management practices constrained more refined analyses of host–pathogen interactions.

## Conclusions

This study estimated a pooled coinfection rate of porcine epidemic diarrhea virus (PEDV) with other pathogens of 12%. A total of 121 distinct coinfection types were identified, with double infections accounting for the majority (83.47%), among which the PEDV-PDCoV combination was the most prevalent. Subgroup analyses further indicated that farm size and coinfection type were the primary contributors to heterogeneity in pooled coinfection rates. Marked spatiotemporal heterogeneity in PEDV infection patterns was observed across China. The eastern and northwestern regions exhibited relatively high coinfection rates with complex pathogen combinations, whereas the central-southern region showed the lowest infection rate, consistent with more effective control measures. In contrast, the northern and southwestern regions displayed stable or dynamically evolving epidemic characteristics. Collectively, these findings have important implications for the prevention and control of PEDV. Future multi-centre, large-scale longitudinal studies integrating pathogen molecular characteristics and host immune data are warranted to refine surveillance strategies and mitigate the impact of PEDV coinfections.

## Data Availability

All data generated or analyzed during this study are included in this published article. The datasets used in the current study are available from the corresponding author upon reasonable request.

## Abbreviations

PEDV: Porcine epidemic diarrhea virus
PRRSV: Porcine reproductive and respiratory syndrome virus
BVDV: Bovine viral diarrhea virus
PDCoV: Porcine deltacoronavirus
PoRV: Porcine rotavirus
TGEV: Transmissible gastroenteritis virus
PCV: Porcine circovirus
PKV: Porcine kolovirus
SADS-CoV: Swine acute diarrhea syndrome coronavirus
PSV: Porcine sapelovirus
GARV: Group A rotavirus
PEAV: Porcine enteric alphacoronavirus
Bhyo: Brachyspira hyodysenteriae
PED: Porcine epidemic diarrhea
PCLV: Porcine circovirus-like virus
PoBuV: Porcine bufavirus
PBoV: Porcine bocavirus
PoRVA: Porcine rotavirus A
EV-G: Enterovirus G
EV: Enterovirus
PKoV: Porcine kobuvirus
PAstV: Porcine astrovirus
PRV: Pseudorabies virus
PSaV: Porcine sapovirus
SaV: Sapovirus
LI: Lawsonia intracellularis
